# The Performance of Three Systems of Glycated Hemoglobin Measurements Among Patients With Sickle Cell Trait in Basrah, Iraq

**DOI:** 10.7759/cureus.77374

**Published:** 2025-01-13

**Authors:** Rafid F Al-Naseri, Nassar T Alibrahim, Sadeq K Al-Salait, Abbas A Mansour

**Affiliations:** 1 Endocrinology and Diabetes, Faiha Specialized Diabetes, Endocrine and Metabolism Center, Basrah, IRQ; 2 Pathology and Laboratory Medicine, Al-Zahraa College of Medicine, Basrah, IRQ

**Keywords:** basrah, diabetes mellitus, endocrinology, hba1c, sickle cell trait

## Abstract

Background

Hemoglobin variants may cause mismanagement of diabetes resulting from false glycated hemoglobin (HbA1c) results. The aim of this study was to compare the results obtained from three different HbA1c assay systems among patients with sickle cell trait (SCT) at Faiha Specialized Diabetes, Endocrine, and Metabolism Center (FDEMC) in Basrah.

Methods

A cross-sectional observational study was done in FDEMC in Basrah on patients with established diagnoses of diabetes mellitus. All samples were analyzed at FDEMC laboratory by using three different systems: Roche Cobas Integra Gen.2 (COBAS INTEGRA^®^ 400 plus analyzer immunoassay) (Roche Diagnostics, Indianapolis, IN), a turbidimetric inhibition immunoassay (TINIA), Bio-Rad Variant II Turbo (Bio-Rad, Hercules, CA) Ion exchange HPLC method, and Bio-Rad D-10 (A1c program) (Bio-Rad, Hercules, CA) Ion exchange HPLC method.

Results

We enrolled 139 persons with diabetes and SCT compared with 70 controls with diabetes and no SCT. A significant difference in the mean HbA1c levels between Roche Cobas Integra Gen.2 and Bio-Rad Variant II Turbo in comparison with Bio-Rad D-10 (A1c program) across all strata of HbA1c in SCT. The highest difference was -0.5% in the stratum of HbA1c 7 to 9% group in Roche Cobas Integra Gen.2, while it was +0.8% in the stratum of HbA1c less than 7% in the SCT group. For the control group, the highest difference was -1.3%, seen in the stratum of HbA1c, more than 9% in the Roche Cobas Integra Gen.2, while the highest difference in the Bio-Rad Variant II Turbo was +0.5% in the same stratum. In both groups, the results of HbA1c were mostly higher in the Bio-Rad Variant II Turbo and lower in Roche Cobas Integra Gen.2.

Conclusion

Roche Cobas Integra Gen.2 and Bio-Rad Variant II Turbo methods are not preferred to be used in HbA1c estimation in areas where SCT is prevalent. Diagnosing or follow-up of glycemic control in patients with SCT needs critical reappraisal because of the limitation of methods used to measure HbA1c in Basrah.

## Introduction

Since its acceptance in 1993 with the Diabetes Control and Complications Trial (DCCT), the determination of glycated hemoglobin (HbA1c) as an observer of the glycemic standing of diabetic patients has surmounted such a high tier of analytical superiority that it is presently advocated as well to be utilized for the diagnosis of diabetes [1,2].

The American Diabetes Association (ADA) recommends performing the HbA1c test at least twice yearly in diabetic patients who have stable blood glucose levels and four times a year in those with changing treatment or have uncontrolled blood glucose levels, so HbA1c testing is crucial to provide more possibilities for timely intervention [3]. At this time, around 100 methods are available in the market worldwide for HbA1c detection with different mechanisms for estimation [4].

Methods used for HbA1c estimation are immunoassay, ion-exchange high-performance liquid chromatography (HPLC), boronate affinity HPLC, enzymatic assays, and capillary electrophoresis [5]. It is well known that there are many obstacles accompanying HbA1c estimation; depending on the patient’s population for a particular laboratory, this should be a significant concern [6]. In managing diabetic patients, the physician should be aware of hemoglobin variants that pose a challenge to HbA1c determination methods because hemoglobin variants may cause mismanagement of diabetes resulting from false HbA1c results [4,7].

One of these hemoglobin variants is Hb S, which represents the result of a genetic defect in the β globulin gene, resulting in the substitution of glutamic acid by valine at position six of β chain [8]. HbA1c estimation depends on hemoglobin with normal structure, so the presence of Hb S may interfere with HbA1c estimation in some bioassays for a variety of reasons [6].

National Glycohemoglobin Standardization Program (NGSP) suggested that the above five methods of measuring HbA1c should not have interference by the presence of Hb S [9], but in Black Americans with sickle cell trait (SCT) causes lower HbA1c compared with fasting and postprandial glucose estimation than those without SCT properly due to other mechanism and not due to the presence of SCT only. Furthermore, SCT could have an effect on diabetes complications, which we do not know yet, and it is no longer considered a benign condition because of the complications that it may cause even without diabetes [10-12].

Since we have a high frequency of diabetes in Basrah, estimated at 19.7% [13], and a high frequency of Hb S, which reached 16% in Abu Al-Khasib District, in the southern part of Basrah [14], so there is a dilemma of a high chance of occurrence of both diabetes and presence of Hb S.

This study is designed to compare the results obtained from three different HbA1c assay systems among patients with SCT at the Faiha Specialized Diabetes, Endocrine, and Metabolism Center (FDEMC) in Basrah.

## Materials and methods

This was a cross-sectional observational study. The participants were selected from FDEMC from February to October 2019. Anthropometric measurements were done for patients, including weight and height, with the corresponding calculation of their body mass indices (BMIs). Basic metabolic panel, including lipid profile, renal function tests, and liver function tests, were done.

The study included 290 with established diagnoses of diabetes mellitus (DM), regardless of the type of diabetes, from both sexes and at any age, whether on oral anti-diabetic medication or on insulin, after obtaining informed verbal consent. We have included patients with HbA1c less than 7%, between 7% and 9%, and more than 9%. One hundred thirty-nine of them were already diagnosed with SCT by using the HPLC system [15] and participated as cases, while the remaining 70 participants, who had no hemoglobinopathy based on their clinical and laboratory diagnoses, were the controls.

Exclusion criteria

All pregnant women, anemic patients (hemoglobin levels <12.0 g/dL in females and <13.0 g/dL in males), and individuals with renal failure (estimated glomerular filtration rate (eGFR) <90 mL/minute/1.73 m^2^), hyperbilirubinemia, and hypertriglyceridemia were excluded.

Blood sampling

A 3 mL whole blood sample in ethylenediaminetetraacetic acid (EDTA) vacutainer was collected through venipuncture from each patient for HbA1c analysis.

HbA1c analysis

**Figure 1:**
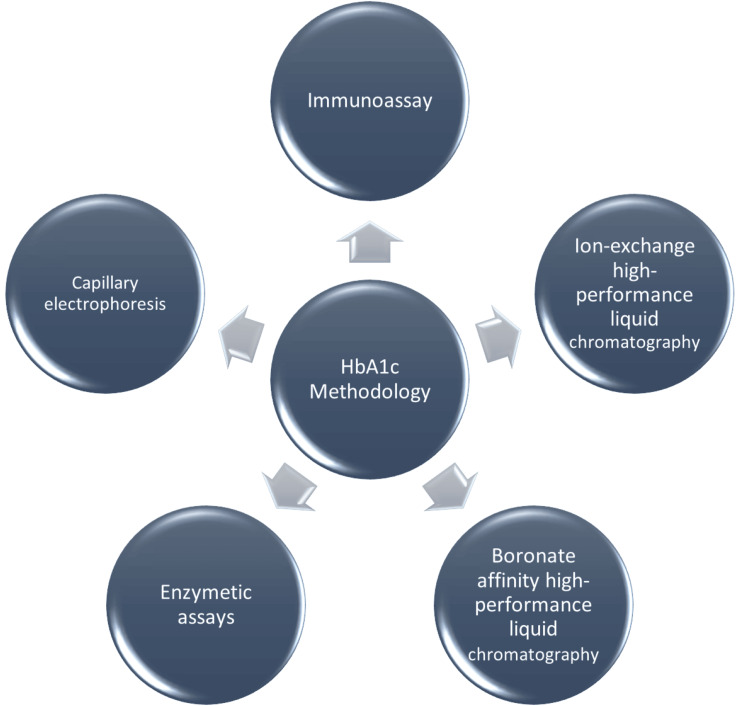
Methodology of HbA1c analysis available

All samples were analyzed at FDEMC laboratory by using three different systems (Figure 1): Roche Cobas Integra Gen.2 (COBAS INTEGRA® 400 plus analyzer immunoassay) (Roche Diagnostics, Indianapolis, IN), a turbidimetric inhibition immunoassay (TINIA), [16] Bio-Rad Variant II Turbo (Bio-Rad, Hercules, CA) Ion exchange HPLC method [17] and Bio-Rad D-10 (A1c program) (Bio-Rad, Hercules, CA) Ion exchange HPLC method [18]. All these systems were used according to the manufacturers’ instructions; the HbA1c values were measured on the same day of sampling.

To compare the readings obtained from the Roche Cobas Integra Gen.2 and Bio-Rad Variant II Turbo methods with the readings obtained from Bio-Rad D-10 (A1c program), we studied the mean difference (deviation) from the results of the Bio-Rad D-10 (A1c program), and how many reading were above or below the standard method; this will help give us an idea about which device (the Bio-Rad Variant II Turbo or the Roche Cobas Integra Gen.2) tend to give higher readings or lower readings than the reference device (the Bio-Rad D-10).

Statistical analysis

All the analyses were performed using the IBM SPSS (IBM SPSS Statistics for Windows, IBM Corp., Version 23.0, Armonk, NY). For continuous variables, we used mean ± standard deviation, while categorical variables were expressed in the form of a number (percentage). A Student t-test was used to compare between means. Paired t-test was used to compare the means of HbA1c obtained from different methods. The chi-square test was used to compare categorical variables. A P-value of less than 0.05 was a cutoff for significance.

Ethical approval and consent

The study design was approved by the ethics committee at FDEMC under reference number 56/35/22, and the center authorities agreed to review the patient's data. Upon registration at the center, all patients included in this study consented to the use of their clinical information for research purposes.

By using a level of significance of 0.05 and a power of 80%, and by using a level of difference of 0.5%, and with the use of our lab data, we have extracted a standard deviation of HbA1c, and it was found to be around 1.4. The calculated sample size according to the formula "n=((Z_α/2_+Z_β_)/ES)^2^ " was 62. We enrolled 70 patients without SCT as controls, and since we were expecting a higher variability of HbA1c in the SCT group (due to the effect of the hemoglobinopathy on the measurement of HbA1c), we doubled the number and enrolled 139 patients.

## Results

The general characteristics of the participants are shown in Table 1. Of the 209 participants with DM in this study, 139 (66.5%) were with SCT, while 70 (32.5%) were controls, and the mean age was 51.09 ± 14.4 years for the SCT group and 50.24 ± 12.4 for the controls; 44.6% were males in the SCT group, while they were 48.6% in the controls, with mean of Hb S 32.4 ± 4.7 in SCT group. The mean BMI in the SCT group and control group was 29.6 ± 5.71 and 29.2 ± 6.60, respectively. Additionally, the duration of diabetes, being on insulin or not, and the duration of insulin therapy were not significantly different between both groups. The mean HbA1c using the three methods in between the two groups was matched.

**Table 1:** General characteristics of the study participants BMI: body mass index, DM: diabetes mellitus, SCT: sickle cell trait Data are shown as mean ± SD or n (%).

	SCT (N = 139)	Control (N = 70)	P-value
Age (years)	51.09 ± 14.4	50.24 ± 12.4	0.677
Gender (males, n (%))	62(44.6)	34(48.6)	0.346
Duration of DM (months)	81.24 ± 7.1	96.15 ± 12.0	0.259
On insulin (yes, n (%))	49(35.3)	27(38.6)	0.374
Duration of insulin (months)	53.2 ± 61.7	46.6 ± 49.8	0.635
BMI (kg/m²)	29.6 ± 5.71	29.2 ± 6.6	0.672
Bio-Rad D-10 (A1c program)	8.9 ± 2.5	9.4 ± 3.3	0.271
Roche Cobas Integra Gen.2	8.7 ± 2.6	8.6 ± 2.8	0.871
Bio-Rad Variant II Turbo	9.7 ± 2.6	9.9 ± 3.4	0.673
Hb S %	32.4 ± 4.7	-	-

Table 2 shows the comparison between the mean HbA1c from different methods within the same group. A significant difference in the mean HbA1c levels between Roche Cobas Integra Gen.2 and Bio-Rad Variant II Turbo in comparison with Bio-Rad D-10 (A1c program) across all strata of HbA1c in SCT. The highest difference was -0.5% in the stratum of HbA1c 7 to 9% group in Roche Cobas Integra Gen.2, while it was +0.8% in the stratum of HbA1c less than 7% in the SCT group.

**Table 2:** Comparison of mean HbA1c according to the levels of HbA1c with Bio-Rad D-10 (A1c program) as reference HbA1c: glycated hemoglobin * A P-value of less than 0.05 was considered the level of significance. Data are shown as mean ± SD.

SCT N = 139			HbA1c < 7% N = 34	HbA1c 7-9% N = 47	HbA1c > 9% N = 58	Total mean HbA1c%
	Roche Cobas Integra Gen.2 vs. Bio-Rad D-10 (A1c program)	Integra	5.6 ± 0.5	7.6 ± 0.7	11.4 ± 1.8	8.7 ± 2.7
		Bio-Rad D-10	5.9 ± 0.6	8.1 ± 0.5	11.6 ± 1.5	9.0 ± 2.6
		Difference	-0.2	-0.5	-0.2	-0.3
		P-value	<0.001*	<0.001*	0.024*	<0.001*
	Bio-Rad Variant II Turbo vs. Bio-Rad D-10 (A1c program)	Variant II Turbo	6.7 ± 0.7	8.8 ± 0.8	12.3 ± 1.7	9.8 ± 2.6
		Bio-Rad D-10	5.9 ± 0.6	8.1 ± 0.6	11.6 ± 1.5	9.0 ± 2.6
		Difference	0.8	0.7	0.7	0.8
		P-value	<0.001*	<0.001*	<0.001*	<0.001*
Controls N = 70			HbA1c < 7% N = 24	HbA1c 7-9% N = 12	HbA1c > 9% N = 34	Total mean HbA1c%
	Roche Cobas Integra Gen.2 vs. Bio-Rad D-10 (A1c program)	Integra	5.8 ± 0.6	7.4 ± 0.8	11.1 ± 1.8	8.6 ± 2.8
		Bio-Rad D-10	6.0 ± 0.7	8.00 ± 0.6	12.4 ± 2.1	9.4 ± 3.3
		Difference	-0.2	-0.6	-1.3	-0.8
		P-value	<0.001*	<0.001*	<0.001*	<0.001*
	Bio-Rad Variant II Turbo vs. Bio-Rad D-10 (A1c program)	Variant II Turbo	6.4 ± 0.8	8.5 ± 0.9	12.9 ± 2.2	9.9 ± 3.4
		Bio-Rad D-10	6.0 ± 0.7	8.0 ± 0.6	12.4 ± 2.1	9.4 ± 3.3
		Difference	0.4	0.5	0.5	0.5
		P-value	<0.001*	0.007*	0.008*	<0.001*

For the control group, the highest difference was -1.3%, seen in the stratum of HbA1c, more than 9% in the Roche Cobas Integra Gen.2, while the highest difference in the Bio-Rad Variant II Turbo was +0.5% in the same stratum. In both groups, the results of HbA1c were mostly higher in the Bio-Rad Variant II Turbo and lower in Roche Cobas Integra Gen.2.

Table 3 shows how much the values of the HbA1c measurement obtained from the Roche Cobas Integra Gen.2 and the Bio-Rad Variant II Turbo methods were deviated from the readings obtained from the Bio-Rad D-10 (A1c program) in comparison between SCT and the control group. Regarding the Bio-Rad Variant II Turbo method, although the mean deviation was not significantly different, the number of readings with positive deviations (readings were higher in the Bio-Rad Variant II Turbo method) were significantly higher in the SCT group (97.8%) in comparison to the control group (81.2%); on the other hand, the number of the readings with a negative deviation (readings were lower in the Bio-Rad Variant II Turbo) were higher in the control group (18.8% versus 2.2%), which means that the difference in the numbers of deviating readings gave a very marginal effect. Furthermore, in the Roche Cobas Integra Gen.2 method, the absolute difference between the reading of HbA1c obtained from the Bio-Rad Variant II Turbo method was significantly lower in the SCT group, and this is mainly due to more readings with negative deviation in the SCT group. However, this device gave fewer readings with negative deviations in the SCT group.

**Table 3:** Mean of absolute deviation of readings of the Bio-Rad Variant II Turbo and Roche Cobas Integra Gen.2 from the reading of Bio-Rad D-10 (A1c program) in cases and controls SCT: sickle cell trait * A P-value of less than 0.05 was considered the level of significance. Data are shown as mean ± SD or n (%).

		SCT	Controls	P
Bio-Rad Variant II Turbo	Absolute mean deviation-total	0.78 ± 0.44	0.68 ± 0.64	0.223
	Mean of positive deviation	0.79 ± 0.44	0.73 ± 0.58	0.412
	Mean of negative deviation	-0.23 ± 0.06	-0.53 ± 0.87	0.575
	Number of positive deviations	135 (97.8)	56 (81.2)	<0.001*
	Number of negative deviations	3 (2.2)	13 (18.8)	
Roche Cobas Integra Gen.2	Absolute mean deviation-total	0.46 ± 0.35	0.86 ± 0.82	<0.001*
	Mean of positive deviation	0.35 ± 0.37	0.23 ± 0.21	0.387
	Mean of negative deviation	-0.51 ± 0.33	-0.95 ± 0.84	<0.001*
	Number of positive deviations	35 (25.5)	8 (11.6)	0.014*
	Number of negative deviations	102 (74.5)	61 (88.4)	

## Discussion

The NGSP updates the methods of estimating HbA1c every time to declare the interference with Hb variants [9]. The new NGSP-approved methods of measuring HbA1c overcome most of the factors interfering with the assay, but the effects of race and aging continue to be solved [4,19].

In this study, we found significant differences between HbA1c estimation in both SCT and control using Roche Cobas Integra Gen.2 or Bio-Rad Variant II Turbo if we compare it with Bio-Rad D-10 (A1c program) as the most widely used approved method for hemoglobin A1c estimation. Roche Cobas Integra Gen.2 tends to give mostly lower results, while the Bio-Rad Variant II Turbo gives higher results in comparison to Bio-Rad D-10 (A1c program) in both groups. However, Roche Cobas Integra Gen.2 tends to give even lower readings in the control group. This result is against NGSP recommendations in that all methods should not be affected by the presence of hemoglobin variants, including sickle hemoglobin, the most common hemoglobinopathy in Iraq [9,19].

The reason for this discrepancy is not easily explained now [4]. In 2010, among 3,378 clinical laboratories that participated in a survey for HbA1c estimation, only 4% of them had clinically significant interference by sickle hemoglobin. Even if the HPLC principle of measurement is used, careful inspection of the chromatogram may reveal the aberrant peaks produced by the variant hemoglobin. In high-volume laboratories, the large-volume methods (Roche Cobas Integra Gen.2 and Bio-Rad Variant II Turbo) are preferred, but this must not be at the expense of accuracy and reliability, as we found a considerable difference between the three methods [4].

Whether the differences in these three methods of estimating HbA1c in both SCT and control can be attributed to racial or ethnic background, it needs to be further evaluated [20]. In patients with suspected SCT, the use of another method for testing glycemic control, like fructosamine, glycated albumin, 1,5-anhydroglucitrol (1,5-AG), and continuous glucose monitoring (CGM), should be considered [4]. However, these alternative methods for glycemic control are not well standardized and are under development [12].

The methods used to diagnose diabetes and assess glycemic control in patients with SCT carry a lot of problems. For example, many patients who are newly diagnosed with diabetes are not aware of being carriers for hemoglobin variants or having raised hemoglobin F for being thalassemia or hereditary persistence of fetal hemoglobin (HPFH) carriers or even homozygous [12].

Although some methods using the principles of HPLC to measure HbA1c can detect some hemoglobin variants and hemoglobinopathies, they are not yet approved methods for such diagnosis. As only a 0.5% change in Hba1c is considered clinically significant, the difference between methods is likely to have any meaningful differences [6]. A study from the same center published in 2013 had the same conclusion: the turbidimetric immunoassay method for the estimation of HbA1c in patients with diabetes and sickle cell disease should be avoided [21].

The limitations of this study were mainly the small study sample, the cross-sectional design, and being a single tertiary center study.

## Conclusions

Roche Cobas Integra Gen.2 and Bio-Rad Variant II Turbo methods are not preferred to be used in HbA1c estimation in areas where SCT is prevalent. Roche Cobas Integra Gen.2 mostly gives lower results in comparison to Bio-Rad D-10 (A1c program), while the Bio-Rad Variant II Turbo method tends to give higher results. Diagnosing or follow-up of glycemic control in patients with SCT needs critical reappraisal because of the limitations of methods used to measure HbA1c.

Upon interpreting the result of hemoglobin A1c, it is important to pay attention to the method used, particularly where hemoglobin variants, such as sickle hemoglobin, are prevalent. Glycemic control in patients with SCT needs critical reappraisal when it comes to hemoglobin A1c estimation and its limitations in these settings. Cobas Integra Gen.2 system tends to give generally lower hemoglobin A1c values compared to other methods.
